# Immunotherapy plus chemotherapy versus chemotherapy alone in the first-line treatment for advanced gastric cancer/gastroesophageal junction cancer: a real-world retrospective study

**DOI:** 10.3389/fimmu.2024.1463017

**Published:** 2024-11-07

**Authors:** Qian Xu, Dan Yi, Caiyan Jia, Fanming Kong, Yingjie Jia

**Affiliations:** ^1^ Department of Oncology, First Teaching Hospital of Tianjin University of Traditional Chinese Medicine, Tianjin, China; ^2^ Department of Oncology, Tianjin Cancer Institute of Traditional Chinese Medicine, Tianjin, China; ^3^ Department of Oncology, National Clinical Research Center for Chinese Medicine Acupuncture and Moxibustion, Tianjin, China

**Keywords:** gastric cancer, immune checkpoint inhibitors, chemotherapy, progression-free survival, overall survival

## Abstract

**Background:**

Immunotherapy offers new hope for improved survival in patients with advanced gastric cancer. Although large randomized controlled trials (RCTs) have been conducted to explore the efficacy and safety of first-line immunotherapy plus chemotherapy versus chemotherapy alone for advanced gastric cancer, the results are not completely consistent. And the strict inclusion criteria of RCTs lead to limited extrapolation. Therefore, it is of great significance to continue to conduct real-world studies comparing the clinical efficacy and safety of immunotherapy combined with chemotherapy versus chemotherapy alone in advanced gastric cancer.

**Methods:**

This retrospective study included patients with HER-2 negative, unresectable advanced or recurrent gastric/gastroesophageal junction cancer (GC/GEJC) who received first-line immune checkpoint inhibitors (ICIs) in combination with chemotherapy or chemotherapy alone between January 1, 2018 to May 31, 2023. Progression-free survival (PFS), overall survival (OS), overall response rate (ORR), disease control rate (DCR) and adverse events (AEs) were compared between two groups.

**Results:**

A total of 210 patients were enrolled in the combination treatment group (n=100) and chemotherapy alone group (n=110). After 12 months of follow-up, median PFS (mPFS) was 270 days (95%CI 177.510-362.490) in the chemotherapy alone group and 357 days (95%CI 250.103-463.897) in the combination treatment group (P<0.05). The median OS (mOS) was 14.9 months (95%CI 9.831-17.769) in the chemotherapy alone group and 15 months (95%CI 12.386-17.614) in the combination treatment group (P>0.05). There was no statistically significant difference in ORR between two groups (P=0.050). The DCR was 14.5% in the chemotherapy alone group and 38% in the combination treatment group (P<0.05). Subgroup analyses showed that primary tumor location of GEJC, ECOG PS of 1, without liver metastasis, and chemotherapy plus ICIs were associated with PFS benefit. Cox multivariate analysis showed that only surgery or not was correlated with patients’ prognosis (P<0.05). Most of AEs were grade 1-2 and manageable.

**Conclusions:**

Compared with chemotherapy alone, first-line ICIs combined with chemotherapy in patients with advanced GC/GEJC could greatly prolong PFS, but OS was not significantly improved, and the AEs were manageable.

## 1 Introduction

Gastric cancer (GC) is responsible for over one million new cases in 2020 and estimated 769,000 deaths, ranking fifth for incidence and fourth for mortality globally (1). In China, approximately 358,700 new cases of GC and 260,400 deaths occurred in 2022, which is the third largest number of cancer deaths (2). Currently, systemic therapy for patients with HER-2 negative, unresectable advanced or recurrent gastric/gastroesophageal junction cancer (GC/GEJC) has been dominated by chemotherapy, and the common first-line agents include platinum, fluorouracil and taxane drugs worldwide (3–5). However, the efficacy of these treatments is not ideal, with the median overall survival (mOS) at approximately only 1 year (6).

Attraction 4, Checkmate 649, and Orient 16 have demonstrated a synergistic effect of immune checkpoint inhibitors (ICIs) in combination with chemotherapy in patients with HER-2 negative, unresectable advanced or recurrent GC/GEJC (7–9). Studies have found that chemotherapy can not only kill tumor cells through cytotoxic effects directly, but also promote anti-tumor immune responses by inducing immunogenic cell death (10–12). As of now, several guidelines, such as the Chinese Society of Clinical Oncology, the European Society for Medical Oncology, and the National Comprehensive Cancer Network, suggest that ICIs together with chemotherapy are used as the first-line treatment for patients with advanced GC especially who exhibit a high combined positive score (CPS) (5, 13, 14).

In China, ICIs are frequently applied to treat unresectable advanced or recurrent GC/GEJC. In order to explore the efficacy and safety of ICIs combined with chemotherapy in these patients, here we examined the short-term and long-term outcomes as well as the adverse events (AEs) of patients who received chemotherapy alone or chemotherapy combined with ICIs.

## 2 Materials and methods

### 2.1 Study design and participants

This retrospective study involved patients with HER-2 negative, unresectable advanced or recurrent GC/GEJC. All patients were fully aware of the purpose of this study and expressed informed consent. This study retrospectively analyzed clinical data of patients with advanced GC/GEJC from January 1, 2018 to May 31, 2023 at the First Teaching Hospital of Tianjin University of Traditional Chinese Medicine in China. Survival data were obtained through follow-up.

All patients had histologically or cytologically confirmed HER-2 negative, unresectable advanced or recurrent GC/GEJC; had received at least two cycles of chemotherapy or chemotherapy combined with ICIs; had received at least one efficacy assessment; had baseline Eastern Cooperative Oncology Group (ECOG) performance status (PS) of 0 or 1; and had normal hepatic and renal function. Patients were excluded if they could not tolerate immunotherapy or chemotherapy; or had severe systemic or autoimmune disease; or multiple primary tumors or unknown primary sites; or were HER-2 positive; or had incomplete clinical data.

### 2.2 Study procedures

All patients included in the final analysis received chemotherapy (XELOX or FOLFOX) and a subset of patients combined with ICIs (nivolumab, sintilimab, tislelizumab and camrelizumab) on this basis.

The baseline information below of each patient were collected: age, sex, family genetic history, history of smoking, history of drinking, ECOG PS, primary tumor location, surgery or not, metastatic site, organs with metastases, and chemotherapy regimen. The clinical efficacy was assessed by outcomes of CT or MRI, which was based on the Response Evaluation Criteria in Solid Tumors (RECIST) version 1.1 (15).

### 2.3 Outcomes

The primary endpoint of this study was progression-free survival (PFS), which was estimated from treatment initiation to progression or death. The secondary endpoints included overall survival (OS), which was defined as the duration from treatment initiation to death due to any reason; objective response rate (ORR), which was defined as the proportion of patients with the best overall response of complete response (CR) or partial response (PR); and disease control rate (DCR), which was defined as the proportion of patients with CR, PR, or stable disease (SD). Safety endpoint included evaluation of AEs. AEs were monitored and classified according to the National Cancer Institute Common Terminology Criteria for Adverse Events (version 5.0).

### 2.4 Statistical analysis

In this study, SPSS 27.0 and GraphPad Prism 9.0 were used for statistical analysis and scientific mapping. Descriptive statistics were used for the basic characteristic data. Chi-square test or fisher’s exact test was used to analyze the efficacy and incidence of adverse reactions. PFS and OS were estimated with Kaplan-Meier method, which was expressed with the two-sided 95% confidence intervals (CIs), and the differences between groups were compared by log-rank test, and the two-sided significance level was P=0.05. The ORR and DCR were analyzed with the Chi-square test. Univariate and multivariate analysis were performed using the Cox proportional hazards model, and hazard ratios (HRs) and 95% CIs were calculated. The difference of P<0.05 was statistically significant.

## 3 Results

### 3.1 Baseline characteristics

**Figure 1:**
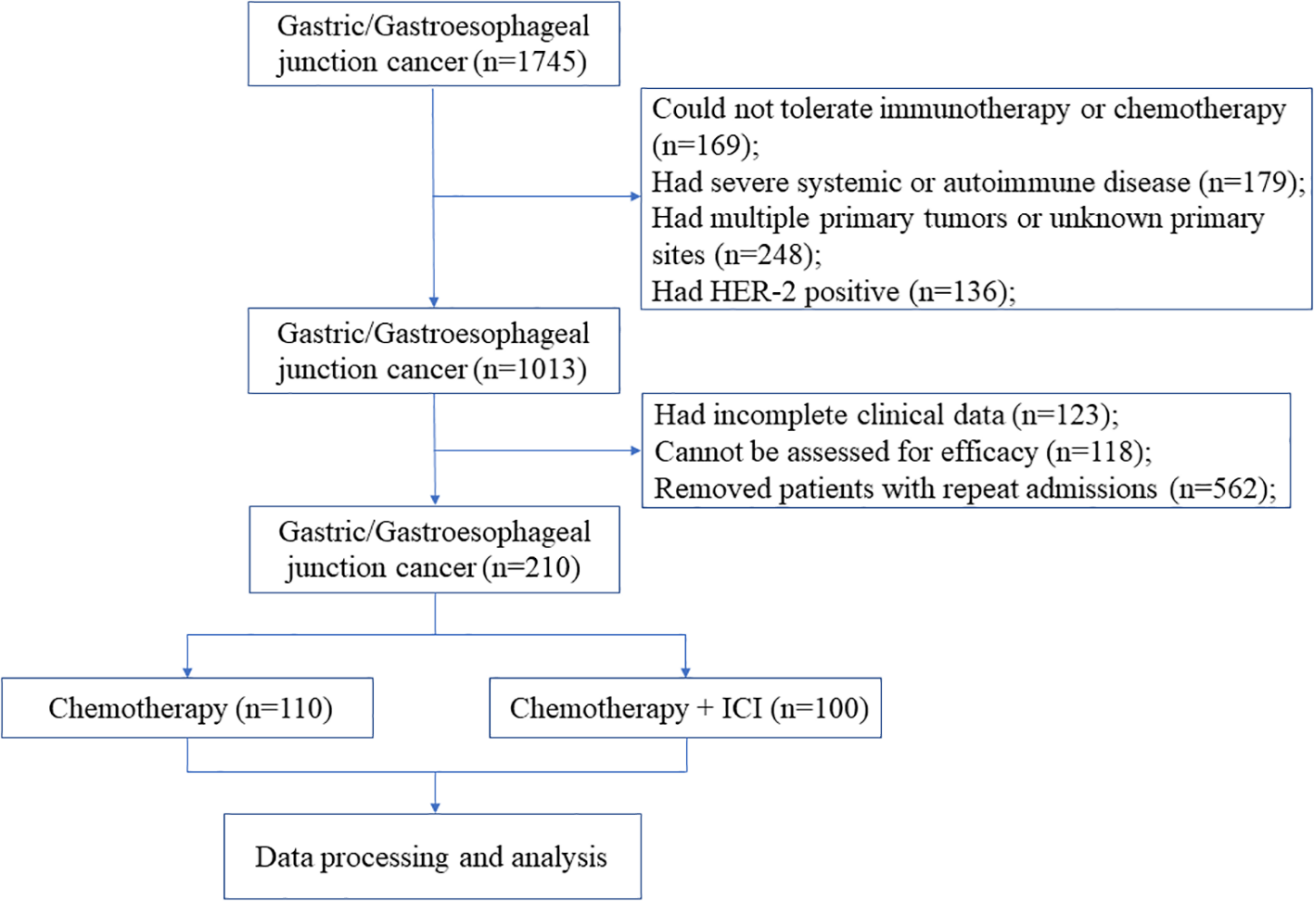
Flow diagram of the study.

After screening 1745 patients according to the inclusion and exclusion criteria described above, we excluded 136 HER-2 positive patients, 169 patients who could not tolerate chemotherapy or immunotherapy, 179 patients who had severe systemic or autoimmune disease, 248 patients with multiple primary tumors or with unknown primary tumor sites, 123 patients without complete clinical data, and 118 patients without evaluable lesions for efficacy. A total of 210 patients were included in the final analysis (**Figure 1**).

The median age of the patients included in the chemotherapy alone group was 64 years old (interquartile range [IQR], 60-70), of which 83 (75.5%) were male, 26 (23.6%) had the family genetic history, 65 (59.1%) had the history of smoking, 52 (47.3%) had the history of alcohol, 101 (91.8%) had a primary tumor site of the stomach, 69 (62.7%) had undergone surgery, half (50%) had two or more sites of tumor metastasis, and the majority of patients (66.4%) received the FOLFOX chemotherapy regimen. The median age of the patients included in the immunotherapy together with chemotherapy group was 66 years old (IQR, 60-73), of whom 66 (66%) were male, 18 (18%) had the family genetic history, 67 (67%) had the history of smoking, 36 (36%) had the history of alcohol, 89 (89%) had a primary tumor site of the stomach, 55 (55%) had undergone surgery, more than half (51%) had two or more sites of metastases, and most (63%) received the FOLFOX chemotherapy regimen. ICIs included nivolumab (6%), sintilimab (70%), tislelizumab (10%), and camrelizumab (14%). All patients had an ECOG performance status of 0-1 (**Table 1**). Because precise PD-L1 CPS values were not available for more than 80% of enrolled patients (PD-L1 CPS ≤1 6 patients, CPS ≥1 10 patients, CPS ≥5 5 patients, and CPS ≥10 2 patients), this metric was not analyzed. Patients who completed first-line therapy without disease progression and tolerable AEs were eligible for maintenance therapy, which consisted of single-agent chemotherapy (S-1 or Capecitabine) with or without immunotherapy.

**Table 1:** Baseline clinical characteristics.

	Chemotherapy	Chemotherapy + ICI	*P* value
Years	64 (60,70)	66 (60,73)	0.725
Sex			0.132
Male	83 (75.5%)	66 (66%)	
Female	27 (24.5%)	34 (34%)	
Family genetic history			0.316
Yes	26 (23.6%)	18 (18%)	
NO	84 (76.4%)	82 (82%)	
Smoking			0.236
Yes	65 (59.1%)	67 (67%)	
NO	45 (40.9%)	33 (33%)	
Drinking			0.098
Yes	52 (47.3%)	36 (36%)	
NO	58 (52.7%)	64 (64%)	
ECOG performance status			0.151
0	54 (49.1%)	59 (59%)	
1	56 (50.9%)	41 (41%)	
Primary tumor location			0.487
GC	101 (91.8%)	89 (89%)	
GEJC	9 (8.2%)	11 (11%)	
Surgery			0.255
Yes	69 (62.7%)	55 (55%)	
NO	41 (37.3%)	45 (45%)	
Metastatic site			0.231
Lymph node	104 (94.5%)	89 (89%)	
Liver	26 (23.6%)	32 (32%)	
Peritoneum	30 (27.3%)	33 (33%)	
Organs with metastases			0.885
1	55 (50%)	49 (49%)	
≥2	55 (50%)	51 (51%)	
Chemotherapy regimen			0.610
FOLFOX	73 (66.4%)	63 (63%)	
XELOX	37 (33.6%)	37 (37%)	
ICIs	–		–
Nivolumab	–	6 (6%)	
Sintilimab	–	70 (70%)	
Tislelizumab	–	10 (10%)	
Camrelizumab	–	14 (14%)	

### 3.2 Efficacy

**Figure 2:**
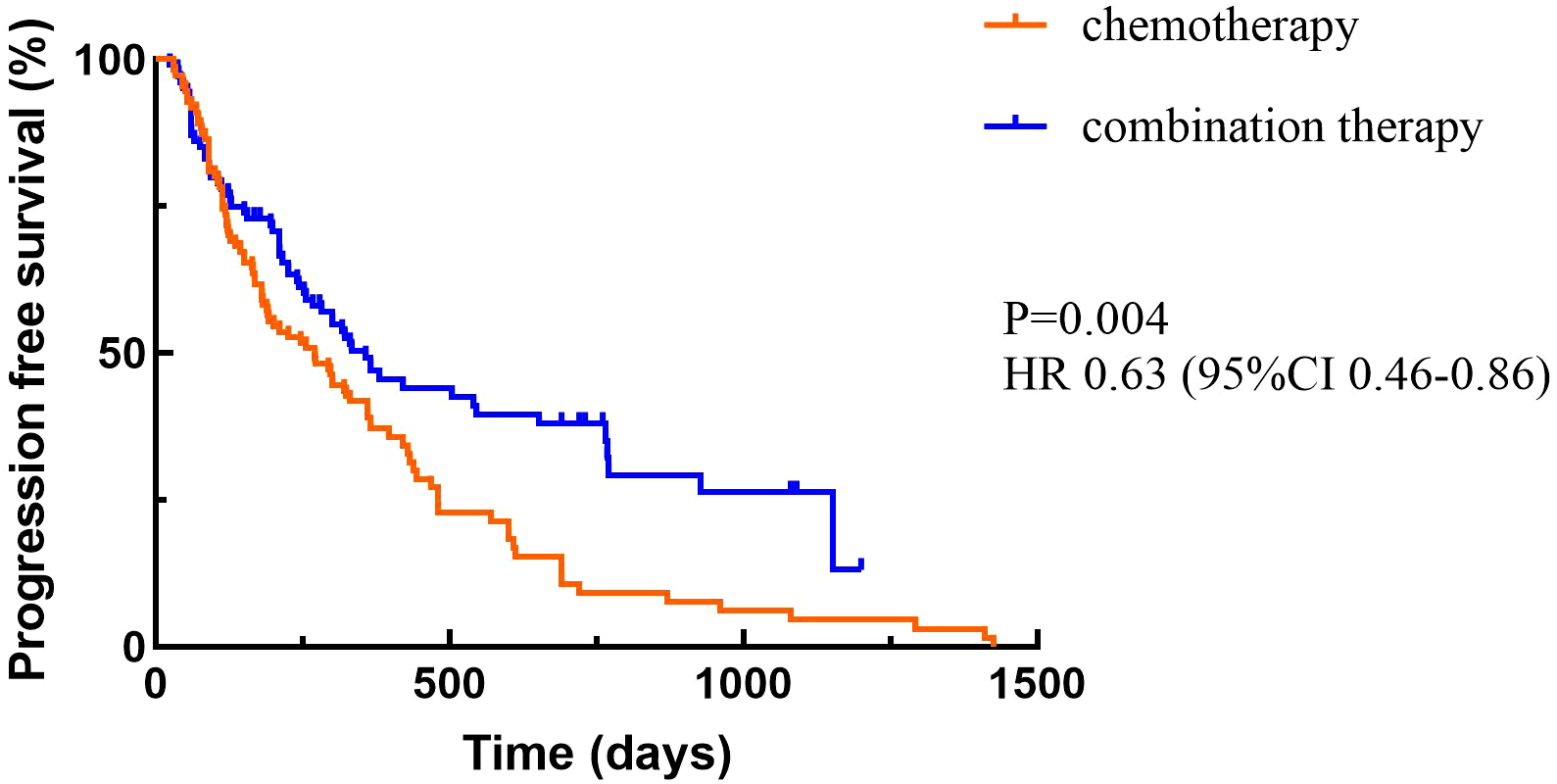
Progression-free survival.

**Figure 3:**
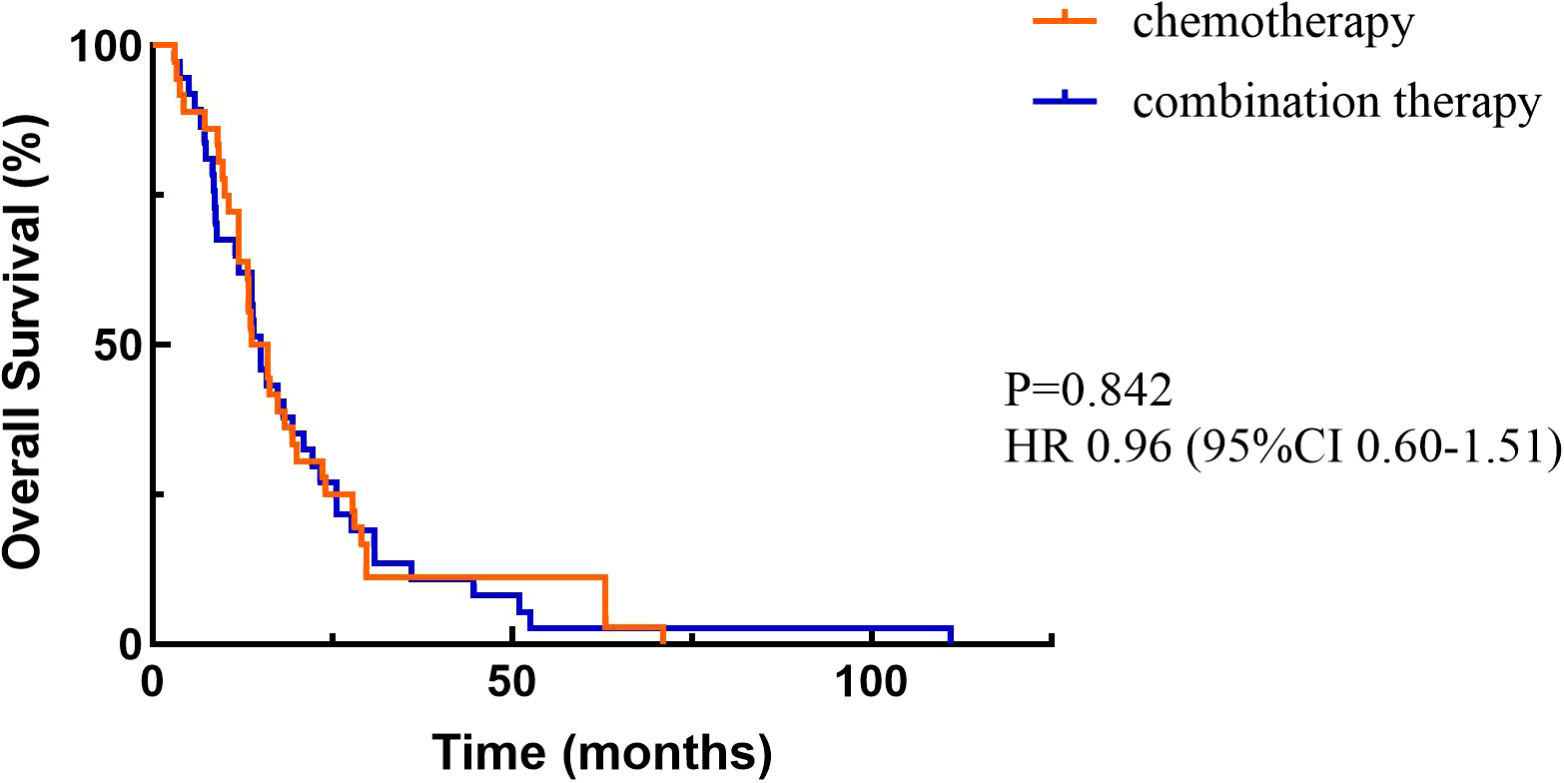
Overall survival.

At the cutoff date, a total of 156 patients out of 210 patients had PD, including 94 in the chemotherapy alone group and 62 in the combination treatment group. Data analysis showed that the median PFS (mPFS) was 270 days (95%CI 177.510-362.490) in the chemotherapy group and 357 days (95%CI 250.103-463.897) in the combination treatment group, and the difference was statistically significant (P<0.05) (**Figure 2**). A total of 36 patients died in the chemotherapy alone group and 37 patients died in the combination treatment group. The median OS (mOS) was 14.9 months (95%CI 9.831-17.769) in the chemotherapy alone group, and 15 months (95%CI 12.386-17.614) in the combination treatment group, and the difference was not statistically significant (P>0.05) (**Figure 3**).

Based on RECIST1.1 criteria, no patients achieved CR and PR, 16 patients achieved SD in the chemotherapy alone group. In the combination treatment group, no patients achieved CR, 4 patients achieved PR, 34 patients achieved SD, and the ORR was 4%. There was no statistically significant difference in ORR between the two groups (P=0.050). The DCR was 14.5% in the chemotherapy alone group and 38% in the combination treatment group. The difference was statistically significant (P<0.05) (**Table 2**).

**Table 2:** ORR and DCR for different treatment regimens.

	Chemotherapy	Chemotherapy + ICIs	*P*
CR	0	0	–
PR	0	4	–
PD	94	62	–
SD	16	34	–
ORR (%)	0%	4%	0.050
DCR (%)	14.5%	38%	0.000

ICI, immune checkpoint inhibitors; CR, complete response; PR, partial response; PD, progression disease; SD, stable disease; ORR, objective response rate; DCR, Disease control rate.

### 3.3 Subgroup analysis

Subgroup analyses showed that primary tumor location of GEJC, ECOG PS of 1, without liver metastasis, and chemotherapy plus ICIs were associated with PFS benefit. The results of univariate analysis of OS showed that age, family genetic history, surgery or not, and organs with metastases were influencing factors (P<0.05), while in further Cox multivariate analysis showed that only surgery or not was correlated with patients’ prognosis (P<0.05). The results of the subgroup analyses of PFS and OS were shown in **Tables 3**, **4**. In addition, we added the analysis of the effect of different ICIs on the efficacy of combination therapy (**Table 5**).

**Table 3:** Univariate and multivariate analyses of PFS.

Features	Univariate analysis		Multivariate analysis	
	HR (95%CI)	*P*	HR (95%CI)	*P*
Age	1.124(0.818,1.545)	0.470	1.201(0.844,1.708)	0.310
Sex	0.690(0.475,1.002)	0.051	0.665(0.423,1.045)	0.077
Primary tumor location	0.394(0.193,0.803)	0.010	0.464(0.220,0.981)	0.044
Family genetic history	0.800(0.538,1.190)	0.271	0.883(0.562,1.387)	0.589
Smoking	1.234(0.897,1.697)	0.197	1.403(0.874,2.254)	0.161
Drinking	0.943(0.685,1.298)	0.719	0.635(0.400,1.009)	0.055
Surgery	1.135(0.825,1.561)	0.437	1.105(0.743,1.645)	0.621
ECOG PS	1.513(1.194,1.917)	0.001	1.344(1.023,1.766)	0.034
Organs with metastases	1.134(0.969,1.328)	0.117	0.815(0.619,1.073)	0.145
Lymphatic metastases	1.016(0.584,1.768)	0.955	0.627(0.325,1.212)	0.165
Liver metastases	0.501(0.357,0.703)	0.000	0.415(0.257,0.669)	0.000
Peritoneal metastases	0.772(0.556,1.073)	0.123	0.699(0.426,1.148)	0.157
BMI	–	0.693	–	0.446
Treatment options	0.620(0.448,0.859)	0.004	0.666(0.466,0.950)	0.025

**Table 4:** Univariate and multivariate analyses of OS.

Features	Univariate analysis		Multivariate analysis	
	HR (95%CI)	*P*	HR (95%CI)	*P*
Age	0.594(0.363,0.970)	0.037	0.896(0.463,1.733)	0.744
Sex	0.870(0.495,1.531)	0.630	0.851(0.405,1.791)	0.672
Primary tumor location	1.210(0.437,3.346)	0.714	1.192(0.352,4.040)	0.778
Family genetic history	0.551(0.315,0.963)	0.036	0.562(0.271,1.165)	0.121
Smoking	1.044(0.652,1.670)	0.859	1.017(0.488,2.116)	0.965
Drinking	0.830(0.518,1.330)	0.440	1.019(0.464,2.241)	0.962
Surgery	1.913(1.177,3.108)	0.009	2.359(1.214,4.581)	0.011
ECOG PS	1.279(0.875,1.870)	0.204	1.415(0.862,2.324)	0.170
Organs with metastases	0.762(0.582,0.999)	0.049	0.604(0.350,1.040)	0.069
Lymphatic metastases	0.572(0.269,1.214)	0.146	0.616(0.239,1.583)	0.314
Liver metastases	0.998(0.619,1.610)	0.995	0.701(0.346,1.421)	0.325
Peritoneal metastases	1.329(0.815,2.167)	0.254	0.720(0.302,1.717)	0.459
BMI	–	0.579	–	0.734
Treatment options	1.048(0.657,1.671)	0.844	1.179(0.647,2.148)	0.591

**Table 5:** Efficacy of different ICIs.

	CR	PR	PD	SD	*P*
Nivolumab	0	0	4	2	0.587
Sintilimab	0	4	40	26	
Tislelizumab	0	0	6	4	
Camrelizumab	0	0	12	2	

### 3.4 Safety

During the treatment, the most common AEs included: leucopenia, neutropenia, anemia, thrombocytopenia, alanine aminotransferase (ALT) increase, aspartate transaminase (AST) increase, creatinine increase, vomiting, peripheral neuropathy, and diarrhea, most of which were of grade 1-2 and manageable (**Table 6**). Any grade anemia was more common in the combination treatment group than in the chemotherapy alone group, but there was no significant difference in the incidence of grade 3 and above. Immunotherapy-related AEs included thyroid dysfunction (2 patients), myocarditis (3 patients), and pneumonitis (2 patients).

**Table 6:** Summary of adverse events.

	Chemotherapy		Chemotherapy + ICI		*P*	
	Any (%)	≥3 grade (%)	Any (%)	≥3 grade (%)	Any	≥3 grade
Leucopenia	20 (18.2%)	0	17 (17%)	1 (1%)	0.822	0.476
Neutropenia	8 (7.3%)	5 (4.5%)	13 (13%)	2 (2%)	0.167	0.521
Anemia	75 (68.2%)	17 (15.5%)	82 (82%)	21 (21%)	0.021	0.297
Thrombocytopenia	17 (15.5%)	5 (4.5%)	18 (18%)	7 (7%)	0.621	0.444
ALT increase	11 (10.0%)	1 (0.9%)	14 (14%)	3 (3%)	0.371	0.547
AST increase	23 (20.9%)	10 (9.1%)	31 (31%)	14 (14%)	0.095	0.264
Creatinine increase	9 (8.2%)	3 (2.7%)	10 (10%)	1 (1%)	0.646	0.682
Vomiting	56 (50.9%)	4 (3.6%)	52 (52%)	5 (5%)	0.874	0.884
Peripheral neuropathy	78 (70.9%)	1 (0.9%)	82 (82%)	3 (3%)	0.059	0.547
Diarrhea	48 (43.6%)	0	43 (43%)	2 (2%)	0.926	0.226
Immunotherapy-related adverse events	–	–	7	0	–	–

ALT, alanine aminotransferase; AST, aspartate transaminase.

## 4 Discussion

Checkmate 649 established the importance of immunotherapy in advanced GC. Recent follow-up data showed (16) that nivolumab plus chemotherapy showed benefit in both OS and PFS compared with chemotherapy alone in both patients with PD-L1 CPS≥5 and all randomized patients. In patients with PD-L1 CPS≥5, mPFS was 8.3 versus 6.1 months (HR=0.71, 95%CI 0.61-0.82) and mOS was 14.4 versus 11.1 months (HR=0.70, 95%CI 0.61-0.81). In all randomized patients, mPFS was 7.7 versus 6.9 months (HR=0.80, 95%CI 0.71-0.89) and mOS was 13.7 versus 11.6 months (HR=0.79, 95%CI 0.71-0.88). Orient 16 (17) demonstrated the population-wide benefit of immunotherapy combined with chemotherapy as a first-line treatment for locally advanced/metastatic GC. The final results showed that in patients with PD-L1 CPS≥5 sintilimab combined with chemotherapy could significantly prolong mPFS (7.7 versus 5.8 months, HR=0.628, P=0.0002) and mOS (19.2 versus 12.9 months, HR=0.587, P<0.0001). In the whole population, mOS was 15.2 versus 12.3 months (HR=0.681, P<0.0001) and mPFS was 7.1 versus 5.7 months (HR=0.638, P<0.0001). Results of rationale 305 (18) also showed that immunotherapy combined with chemotherapy as a first-line treatment can significantly prolong survival in patients with locally advanced unresectable or metastatic GC/GEJC.

Notably, keynote 062 (19) and attraction 4 (7) received partially negative results, which were also RCTs comparing the efficacy and safety of immunotherapy plus chemotherapy with chemotherapy alone, and the addition of immunotherapy did not result in a significant final OS benefit. In this retrospective study, we found that chemotherapy combined with ICIs was effective in improving PFS (mPFS 270 versus 357 days, P<0.05), which is consistent with previous studies. However, there was no significant difference in OS (mOS 14.9 versus 15 months, P>0.05), which we considered that it may be related to the level of CPS expression, mismatch repair status, subsequence lines of treatment and the length of follow-up. Although PD-L1 CPS≥5 has been shown to be a good independent prognostic factor for survival (16–18), interestingly a systematic review found that when ICI was combined with chemotherapy, the correlation between PD-L1 expression and ORR was not obvious. The pooled ORR in PD-L1 negative, PD-L1 CPS ≥1, PD-L1 CPS ≥5, and PD-L1 CPS ≥10 population was 57%, 48%, 60%, and 58%, respectively. It seems that the benefit brought about by the rise in PD-L1 expression was not obvious when ICI and chemotherapy were combined (20). This requires further exploration on the effect of CPS on the efficacy of immunotherapy combined with chemotherapy in advanced GC. In addition, OS was closely related to follow-up time. Because the follow-up of this study was only one year, there may be bias, and we will continue to follow up these patients in the future. None achieved CR or PR in the chemotherapy alone group, compared with only 4 patients of PR in the combination group. There was no statistically significant difference in ORR between two groups (P=0.050). The DCR was 14.5% in the chemotherapy alone group and 38% in the combination treatment group (P<0.05). ORR and DCR can be affected by a variety of factors, including the level of immunity, the type of ICIs used, PD-L1 CPS expression level, tumor characteristics, molecular phenotypes, and performance status of patients. The combination of these factors determined the efficacy of patients receiving immunotherapy in combination with chemotherapy. Subgroup analyses showed that primary tumor location of GEJC, ECOG PS of 1, without liver metastasis, and chemotherapy plus ICIs were associated with patient PFS benefit. Cox multivariate analysis showed that only surgery or not was correlated with patients’ prognosis (P<0.05). Although there was no statistically significant difference in the effect of different ICIs on combination therapy in this study, it is still worth further exploring whether this is related to sample size and regional differences. Most AEs were grade 1-2 and manageable. In this study any grade of anemia was more common in the combination treatment group than in the chemotherapy alone group, but there was no significant difference in the incidence of grade 3 and above. Immunotherapy-related AEs included thyroid dysfunction (2 patients), myocarditis (3 patients), and pneumonitis (2 patients). Although the incidence of grade 3 and above AEs in chemotherapy combined with immunotherapy is low, close attention should be paid to prevent the occurrence of severe immune-related AEs and more in-depth analysis of it could follow to provide targeted remissions. At present, large real-world studies comparing the efficacy and safety of immunotherapy combined with chemotherapy are still needed, and there is an urgent need to study the dominant population and dominant stage of immunotherapy.

In this study, patients were collected according to strict inclusion criteria, and also multivariate analysis was used to control for the impact of confounding factors to minimize error. As this was a retrospective real-world study, clinical data collection was based on the extraction of electronic medical records and patient follow-up, and the data for safety analysis were mainly from medical records, laboratory indicators and imaging tests. The potential bias due to the retrospective, non-randomized design remains a limitation of this study. Many pathology centers, including ours, do not perform routine CPS detection, so the records of PD-L1 CPS expression level in patients were incomplete. And this study is only a single-center study, which has the problem of small sample size. In addition, HER-2 positive patients, who account for 20% of all GC patients (21), were not enrolled in the study, and there is also a clinical need to help these patients improve their survival, so we are conducting further prospective studies on different CPS levels, HER-2 expression levels, microsatellite status, and different ICIs.

## 5 Conclusion

In conclusion, the results of the study showed that in patients with HER-2 negative, unresectable advanced or recurrent GC/GEJC chemotherapy combined with ICIs could greatly prolong PFS, but OS was not significantly improved, and AEs were manageable. The outcomes confirmed the efficacy and safety of immunotherapy in combination with chemotherapy in the real-world setting, which could provide the basis for the standard first-line treatment of these patients.

## Data Availability

The original contributions presented in the study are included in the article/supplementary material. Further inquiries can be directed to the corresponding authors.
